# Influence of water deficit on the induced and constitutive responses of pines to infection by mountain pine beetle fungal associates

**DOI:** 10.1186/1753-6561-5-S7-O29

**Published:** 2011-09-13

**Authors:** Adriana Arango-Velez, Miranda Meents, Jean Linsky, Walid El Kayal, Eri Adams, Leonardo Galindo, Janice Cooke

**Affiliations:** 1 Department of Biological Sciences, University of Alberta, Canada

## Background

The ongoing outbreak of mountain pine beetle (MPB; *Dendroctonus ponderasae* Hopkins) and its associated pathogenic fungi (e.g. *Grosmannia clavigera* [Robinson-Jeffrey and Davidson] Zipfel, de Beer and Wingfield) in western North America has resulted in the loss of more than 13 million hectares of pines since 1999 in British Columbia alone [1]. MPB has principally attacked lodgepole pine (*Pinus contorta* Dougl. ex Loud. var. *latifolia*) in British Columbia. However, since 2006 MPB has spread into northern Alberta, where lodgepole pine hybridizes with jack pine (*Pinus banksiana* Lamb.) [2]. Few studies have compared lodgepole and jack pine defense responses, but given that lodgepole pine and MPB share a co-evolutionary history [1] whereas jack pine is a new host for MPB [2], it is reasonable to expect that differences might exist. Some regions affected by the current outbreak have experienced drought conditions during the last decade. Water deficit can limit carbon assimilation, potentially increasing tree susceptibility to MPB and their symbiotic fungi [3]. We are testing the hypotheses that lodgepole and jack pine defenses against MPB and *G. clavigera* differ, and that water deficit affects these responses.

## Materials and methods

The relationship between water availability and tree defense was evaluated in (1) two year old lodgepole and jack pine seedlings in growth rooms, and (2) ca. sixty year old lodgepole x jack pine hybrids in naturally regenerated, thinned stands. Soil relative water content was monitored using time-domain reflectometry. Seedlings were subjected to watering or water deficit for one week prior to wounding or wounding plus *G. clavigera* inoculation. Mature trees were either watered or water limited via tarps for six weeks before wounding plus *G. clavigera* inoculation. In both experiments, water deficit conditions were maintained throughout the time course.

Tree physiological status was evaluated by measuring gas exchange and stomatal conductance using a LiCor 6400, stem hydraulic conductivity using a low-pressure flow meter and safranin dye xylem perfusion, and HPLC. Defense responses were assessed by lesion measurements histochemistry, and qRT-PCR.

## Results and discussion

Stomatal conductance and photosynthesis significantly decreased under water deficit for both lodgepole and jack pine seedlings, but seedling hydraulic conductivity was not affected. The mild water deficit applied to the mature trees reduced stomatal conductance and photosynthesis, but not significantly.

Stem lesions are a means of killing and compartmentalizing invading organisms [4]. *G. clavigera*-induced lesions developed more slowly in jack pine than lodgepole pine seedlings. Stem hydraulic conductivity decreased in inoculated lodgepole but not jack pine seedlings, likely because of greater tracheid occlusion caused by increased fungal growth and/or resin production in lodgepole pine [5]. Water deficit reduced lesion development rates at early time points in inoculated lodgepole and jack pine seedlings, as well as in mature trees at 5 weeks post-inoculation. Lesion length has been considered an indicator of tree defense capacity [6], with longer lesions reported to reflect increased release of toxic and/or inhibitory substances [7]. Accordingly, we interpret slower lesion development to indicate a slower defense response. Our results suggest (1) more rapid defense responses to *G. clavigera* in the co-evolved lodgepole pine host than in the new jack pine host, and (2) defense responses are slowed by water limitation.

We then examined the effect of water deficit on transcript abundance corresponding to genes classically associated with drought and defense responses. We conducted qRT-PCR transcript abundance profiling of secondary phloem from mature lodgepole x jack pine hybrids. We first profiled four *aquaporin* and five *DREB* genes, families associated with water stress responses. Although the mild water deficit did not significantly alter expression of these genes, expression of one *aquaporin* and one *DREB* decreased in response to *G. clavigera* inoculation. We then profiled five *chitinase* and four *terpene synthase* defense-associated genes. Expression of two *chitinases* was significantly induced by water deficit but not *G. clavigera*. Expression of other *chitinases* significantly increased in response to fungalinoculation, but the response was attenuated by water deficit. Expression of one *terpene synthase* significantly increased with fungal inoculation, but this response was also attenuated under water deficit. In contrast, water deficit increased constitutive expression of another *terpene synthase*. Higher constitutive expression of the *monoterpene synthase* under mild water stress suggests a pre-emptive defense via higher biosynthesis of volatile monoterpenes. Microarray and qRT-PCR analyses of the lodgepole and jack pine seedling experiment are underway.

## Conclusion

Our analyses suggest that defense responses of jack pine differ from those of lodgepole pine. Molecular analyses are underway to further characterize these differences. Both constitutive and induced defense responses are modulated in pines by water deficit, and this response appears to be gene-specific. This study shows evidence of cross talk between the water stress and defense responses of pine trees.
